# Supplementary figures and images for: Neuropeptide Bursicon and its receptor-mediated the transition from summer-form to winter-form of Cacopsylla chinensis
Source: eLife. 2024 Nov 8;13:RP97298. doi: 10.7554/eLife.97298 (PMC11548876; doi:10.7554/eLife.97298)

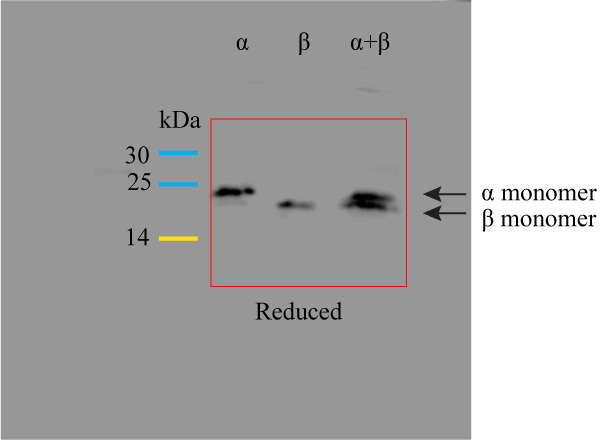

Supplement: Figure 1—source data 1. [file elife-97298-fig1-data1.tif]

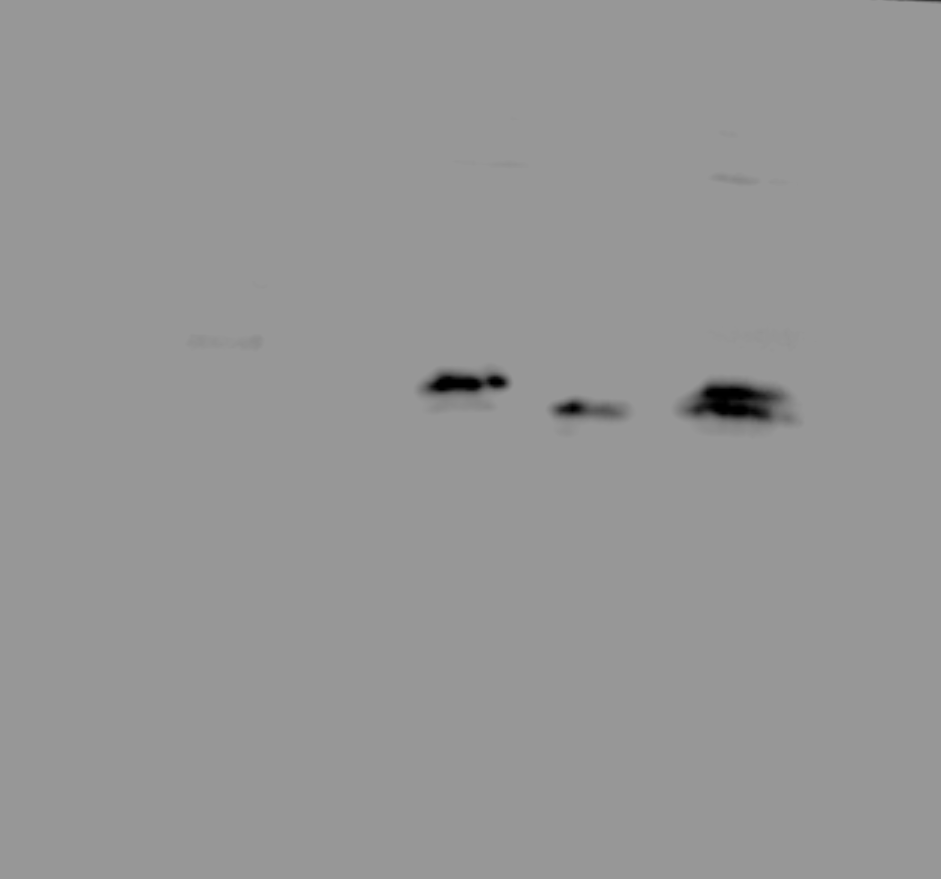

Supplement: Figure 1—source data 2. [file elife-97298-fig1-data2.tif]

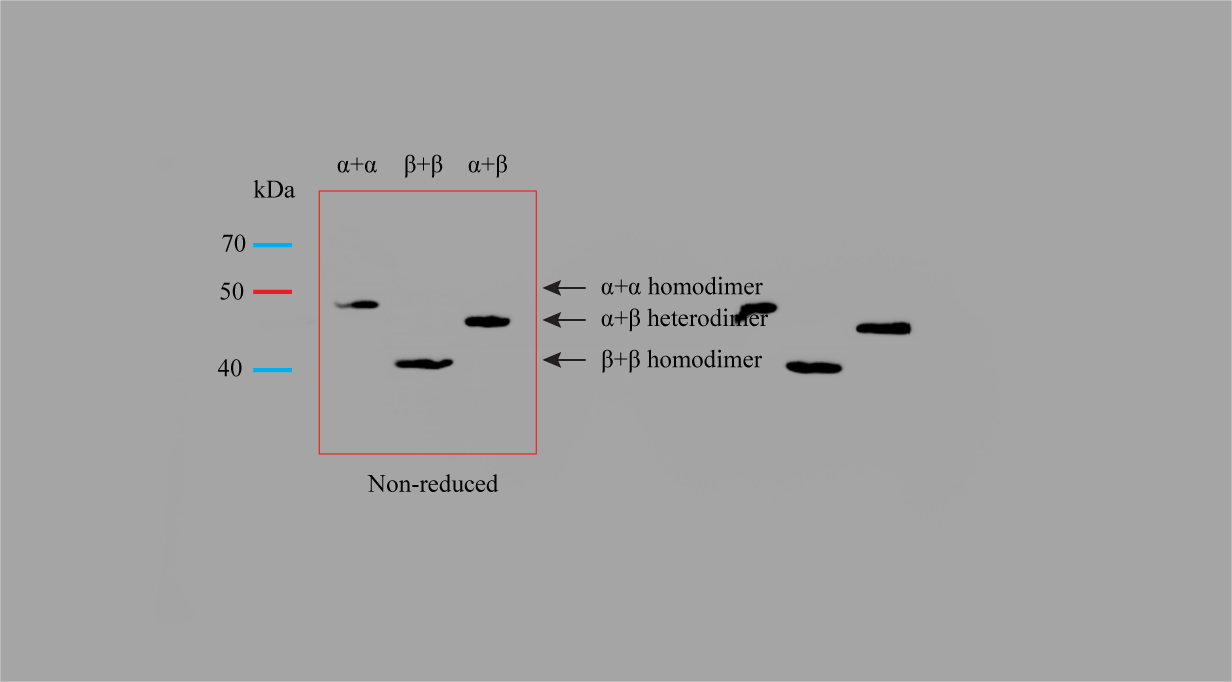

Supplement: Figure 1—source data 3. [file elife-97298-fig1-data3.tif]

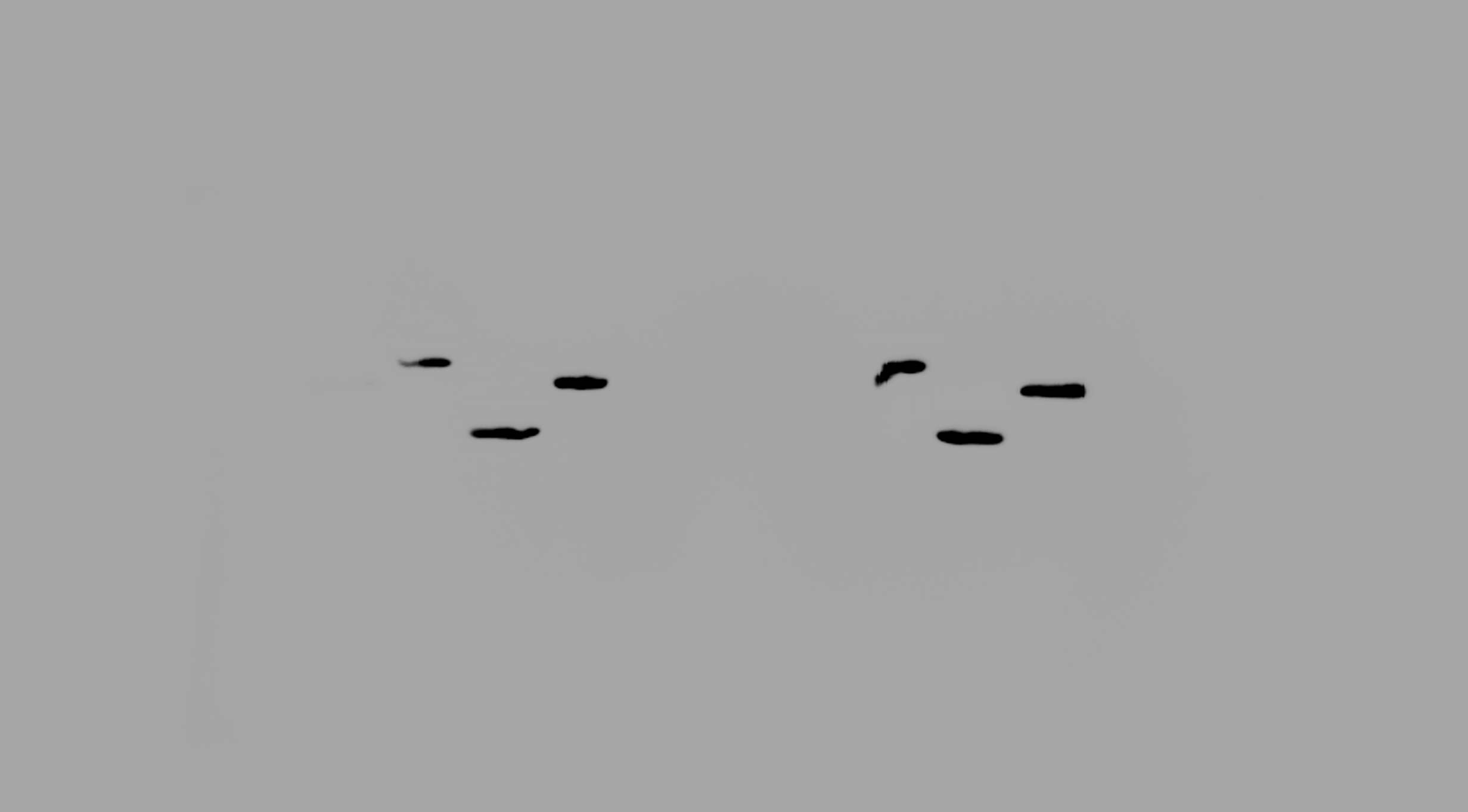

Supplement: Figure 1—source data 4. [file elife-97298-fig1-data4.tif]
