# Supplementary material for: Neuropeptide Bursicon and its receptor-mediated the transition from summer-form to winter-form of Cacopsylla chinensis
Source: eLife. 2024 Nov 8;13:RP97298. doi: 10.7554/eLife.97298 (PMC11548876; doi:10.7554/eLife.97298)
Supplement: Supplementary file 1. — (a) List of primers used in this study. (b) Comparison of pigmentation and cuticle thickness after CcTRPM, CcBurs-a, CcBurs-β, and CcBurs-R knockdown. [file elife-97298-supp1.docx]

**Supplementary File 1**

**Neuropeptide Bursicon and its receptor mediated the** **transition from** **summer-form to** **winter-form of *Cacopsylla chinensis***

Zhixian Zhang^1^, Jianying Li^1^, Yilin Wang^1^, Zhen Li^1^, Xiaoxia Liu^1^, Songdou Zhang^1,2,*^

^1^Department of Entomology and MOA Key Lab of Pest Monitoring and Green Management, College of Plant Protection, China Agricultural University, 100193 Beijing, China

^2^Sanya Institute of China Agricultural University, 572025 Sanya City, Hainan Province, China

^*^**Corresponding author**: Songdou Zhang.

**Email:** zhangsongdou1128@126.com

**Supplementary file 1a. List of primers used in this study.**

| **Gene name and GenBank ID** | **Forward Primer (5'-3')** | **Reverse Primer (5'-3')** | **Product size (bp)** | **Application purpose** |
| --- | --- | --- | --- | --- |
| *CcBurs-α*  (OR488624) | Full-F: CACATGTCGGACGGTATAA | Full-R: GCCACTCAATCTCTTCAGAC | 505 | Full-length cDNA cloning |
|  | RNAi-F1: ggatcctaatacgactcactatagg  AGTGTGTCTTCTGTTACCCCT | RNAi-R1: ggatcctaatacgactcactatagg  CACTAGCCTCTCTTTCTCCACT | 326 | dsRNA synthesis |
|  | qF: TACAAGCATTGAGGAGAG | qR: TCATTCTGACTTGGCAAA | 94 | qPCR |
|  | pcDNA3.1-his-mCherry-F1: ccactagtccagtgtggtggaattc  GGCCGCATCTTCTGGTAAA | pcDNA3.1-his-mCherry-R1: gtttaaacgggccctctagactcgag  CACTTAGTGATGGTGATGGTG | 114 | Reduced gel |
|  | pcDNA3.1-CcBurs-α-his-mCherry-F2: ctagcgtttaaacttaagctt  GCCACCATGTCGGACGGTATAATGAACA | pcDNA3.1-CcBurs-α-his-mCherry-R2: ccacactggactagtggatcc  TTCTGACTTGGCAAAATGTCC | 547 | Reduced gel |
|  | pcDNA3.1-CcBurs-α-his-P2A-mCherry-F3: gctggctagcgtttaaacttaagctt  GCCACCATGTCGGACGGTAT | pcDNA3.1-CcBurs-α-his-P2A-mCherry-R3: tccgcttccggtaccaagctt  GTGATGGTGATGGTGATGGTGGTG | 665 | Reduced gel |
|  | pcDNA3.1-CcBurs-α-his-P2A-mCherry-F1: TGGTGCACGTGGAAGCTTCCAACATTTCAGTCTCATCAGCC | pcDNA3.1-CcBurs-α-his-P2A-mCherry-R1: AGGCAGCTGGCGCGCTCCATGGTGGCAAGCTTAAGTTTAAAC | 6938 | Non-reduced gel |
|  | pcDNA3.1-CcBurs-α-his-P2A-mCherry-F2:  GCTGCTGCTGCTGCTCCCCCTGGTGCACGTGGAAGCTTCC | pcDNA3.1-CcBurs-α-his-P2A-mCherry-R2:  GGGGGAGCAGCAGCAGCAGCAGGCAGCTGGCGCGCTCCAT | 6958 | Non-reduced gel |
|  | Full-F: TTGTACACAAGCAATAATGTCC | Full-R: ACGTCATGAGTGACACAAAC | 446 | Full-length cDNA cloning |
| *CcBurs-β*  (OR488625) | RNAi-F1: ggatcctaatacgactcactatagg  TCCTTTTGTTTGAAATGGGCTCT | RNAi-R1: ggatcctaatacgactcactatagg  GGAAACTTTCTCGACAGCAGT | 336 | dsRNA synthesis |
|  | qF: GTGAGAAGATGGCAACATTAGA | qR: GCGAATAATCTCCACACTTGTA | 78 | qPCR |
|  | pcDNA3.1-P2A-mCherry-F1: tttaaacttaagcttggtacc  GGAAGCGGAGCTACTAACTTCAGCCTG | pcDNA3.1-P2A-mCherry-R1: gctcaccatggtggcggtacc  AGGTCCAGGGTTCTCCTCCACG | 108 | Reduced gel |
|  | pcDNA3.1-CcBurs-β-his-mCherry-F2: ctagcgtttaaacttaagctt  GCCACCATGTCCTTTTGTTTGAAATGGG | pcDNA3.1- CcBurs-β-his-mCherry-R2: ccacactggactagtggatcc  TCGCGAATAATCTCCACACT | 408 | Reduced gel |
|  | pcDNA3.1-CcBurs-β-his-P2A-mCherry-F3: gctggctagcgtttaaacttaagctt  GCCACCATGTCCTTTTGTTTGAAATGG | pcDNA3.1-CcBurs-β-his-P2A-mCherry-R3: tccgcttccggtaccaagctt  GTGATGGTGATGGTGATGGTGGTG | 590 | Reduced gel |
|  | pcDNA3.1-CcBurs-β-his-P2A-mCherry-F1: TGGTGCACGTGGAAGCTTCCGAAAAGGACGAGGCTTGT | pcDNA3.1-CcBurs-β-his-P2A-mCherry-R1:  AGGCAGCTGGCGCGCTCCATGGTGGCAAGCTTAAGTTTAAAC | 6863 | Non-reduced gel |
|  | pcDNA3.1-CcBurs-β-his-P2A-mCherry-F2:  GCTGCTGCTGCTGCTCCCCCTGGTGCACGTGGAAGCTTCC | pcDNA3.1-CcBurs-β-his-P2A-mCherry-R2:  GGGGGAGCAGCAGCAGCAGCAGGCAGCTGGCGCGCTCCAT | 6883 | Non-reduced gel |
| *CcBurs-R*  (OR488626) | Full-F: TTTCATCGTCGTTTGGAGAT | Full-R: CCCGAAAGTATCCCATAGTG | 1758 | Full-length cDNA cloning |
|  | RNAi-F1: ggatcctaatacgactcactatagg  ATCTGACCCAATTGAGGAAC | RNAi-R1: ggatcctaatacgactcactatagg  CCGTCCGGTATGTATTTGAT | 371 | dsRNA synthesis |
|  | orf-F1 | orf-R1 |  |  |
|  | AGGTCTATATAAGCAGAGCTC | AGGTCTATATAAGCAGAGCTC | 1811 | Amplification of |
|  | ATGAAGAAAACGTCATTTTTAATA | ATCAATACCCGGAGTCAAATGA |  | orf sequence |
|  | qF: CCTCAGCAAACTACAAGTCT | qR: GCCCAGGTTCAAATCTTCTA | 103 | qPCR |
|  | 3'UTR-Full-F1: tctagttgtttaaacgagctc  CGCTTGGCATAGTTCCATAT | 3'UTR-Full-R1: cctgcaggtcgactctaga  GGTACCTAACCTACCCAGAT | 599 | Application of  3'UTR Full sequence |
|  | 3'UTR-Mut-F1: tctagttgtttaaacgagctc  TTGTTCCCACTTCTTCCTGGAA | 3'UTR-Mut-R1: cctgcaggtcgactctaga  GGTACCTAACCTACCCAGAT | 382 | Application of  3'UTR mutant sequence |
| *CcTRPM*  (OQ658558) | RNAi-F: ggatcctaatacgactcactatagg  GGTACCTTCGTATCCTCAAC | RNAi-F: ggatcctaatacgactcactatagg  TGGCATTGATCTCCATGAAA | 472 | dsRNA synthesis |
|  | qF: ACAATGCATTCTTCTTGACC | qR: GACTTTGTACACTGGGGGTA | 141 | qPCR |
| *EGFP*  (ACY56286) | RNAi-F: ggatcctaatacgactcactatagg  ACTCCAGCAGGACCATGTGATC | RNAi-F: ggatcctaatacgactcactatagg  CCTGAAGTTCATCTGCACCAC | 596 | dsRNA synthesis |
| *CcTre1*  (OQ734934) | qF: GGAACTCCCTCCTCTATGTT | qR: CCAATAGTCAGCCAATCTGT | 147 | qPCR |
| *CcCHS1*  (OQ658570) | qF: AGAAGAGAAGAAACAGCAGG | qR: TAGTGTCCAATCGTTTCTCC | 233 | qPCR |
| *Ccβ-actin*  (OQ658571) | qF: CGTATGCAGAAGGAAATCAC | qR: AGATCCACATCTGTTGGAAG | 139 | qPCR |
| miR-6012 | qF: TTCGGCGATGAGATCAGCCAGT | - | - | qPCR |
| U6 | qF:AGGATGACACGCAAAATCGT | - | - | qPCR |

**Supplementary file 1b. Comparison of pigmentation and cuticule thickness after *CcTRPM*, *CcBurs-a*, *CcBurs-β*, and *CcBurs-R* knockdown.**

|  | **dsEGFP** | **dsCcTRPM** | **dsCcBurs-a** | **dsCcBurs-β** | **dsBurs-R** |
| --- | --- | --- | --- | --- | --- |
| Pigmentation  (absorbance at 300nm) | 0.8499 ± 0.0532a | 0.1779 ± 0.0059b | 0.1846 ± 0.0203b | 0.1931 ± 0.0309b | 0.1410 ± 0.0131b |
| Cuticule thickness  (μm) | 3.39 ± 0.20a | 1.75 ± 0.09b | 1.44 ± 0.12b | 1.54 ± 0.15b | 1.34 ± 0.13b |
